# Computational and experimental analysis of oleanolic acid as an allosteric activator of SIRT1

**DOI:** 10.55730/1300-0527.3786

**Published:** 2025-12-07

**Authors:** Emrah SARIYER, Eda DOKUMACIOĞLU, Hatice İSKENDER

**Affiliations:** 1 Medical Laboratory Techniques, Vocational School of Health Services, Artvin Çoruh University, Artvin, Turkiye; 2 Department of Nutrition and Dietetics, Faculty of Health Sciences, Artvin Çoruh University, Artvin, Turkiye

**Keywords:** Molecular dynamics simulations, oleanolic acid, sirtuin 1, resveratrol

## Abstract

Sirtuin 1 (SIRT1) is a member of the sirtuin protein family and the biological effects of SIRT1 encompass various areas such as the ageing process, metabolism regulation, and cellular stress response. SIRT1 activators are compounds that enhance the activity of SIRT1 enzyme. These activators can be natural or synthetic compounds and have the potential to provide health benefits by augmenting the biological effects of SIRT1. Oleanolic acid (OA) is a triterpenoid compound that has antiinflammatory, antioxidant, and glucose and lipid metabolism-regulating properties naturally found in many plants. The aim of the present study was to investigate the effect of OA on SIRT1 activation and its underlying mechanism. SIRT1 activity was determined by ELISA in rat serum and liver and kidney tissue. SIRT1 activity was significantly higher in the OA-treated rats compared to the control group. Moreover, through the use of computational methods, the study examined the potential of OA as an allosteric compound for SIRT1. The findings revealed that OA demonstrated a greater affinity for the allosteric site than resveratrol did and may lead to a similar increase in SIRT1 activity. The findings of both experimental and computational studies were complementary, which led to the conclusion that OA serves as a positive regulator of SIRT1 activity.

## 1. Introduction

In recent years, herbs have been extensively utilized as alternative or supplementary treatments for chronic diseases, particularly in developing nations. The established therapeutic benefits of plants have intrigued researchers, leading them to explore bioactive compounds found in plants and their derivatives [1]. This interest focuses on identifying and understanding the active substances that contribute to these medicinal effects. Various phytochemicals with biological activities against chronic diseases have been identified in the literature, and these substances have been increasingly utilized for their activating or inhibitory effects [2].

Oleanolic acid (OA), a pentacyclic triterpenoid, is a natural product isolated from various food and medicinal plants [3]. It has gained significant attention in research due to its potential therapeutic properties and biological activities. OA possesses antioxidant functions, which means it can assist in counteracting damaging free radicals within the biological system. This antioxidant role may enhance its potential protective role against oxidative stress-related diseases, including certain cancers and cardiovascular diseases [4,5].

Sirtuin 1 (SIRT1) is part of the sirtuin family and functions as a type III histone deacetylase, which is crucial for epigenetic modifications in humans [6]. SIRT1 acts as a regulatory link between cellular metabolic conditions and gene expression. It is essential for various processes, including DNA repair, programmed cell death, differentiation of muscle and fat cells, neuron formation, and mitochondrial synthesis [7,8]. SIRT1 is a key protein involved in the regulation of various cellular processes, including glucose and lipid metabolism, autophagy, circadian rhythm, apoptosis, stress response, DNA repair, activation of transcription factors, and maintenance of cellular homeostasis [9,10]. In particular, it can exert deacetylation effects toward various target proteins, enhancing cellular energy production and optimizing mitochondrial functions [11]. SIRT1 may enhance insulin sensitivity and play a protective role against the development of metabolic diseases. Therefore, studies about SIRT1 are crucial for understanding metabolic disorders such as diabetes and obesity [12,13]. SIRT1 in various metabolic tissues is controlled by key biological modulators, making it a focus for numerous SIRT1-activating compounds (STACs) [14]. Sirtuin activators are generally natural or synthetic compounds that can enhance the activity of sirtuins, thereby regulating cellular functions. Some of these activators can be found naturally in certain foods. The most widely known SIRT1 activator is resveratrol [15].

Growing evidence highlights the role of OA in modulating SIRT1 activity, indicating it to be a promising therapeutic candidate in conditions associated with neuroinflammation and oxidative damage [16]. The interaction between OA and SIRT1 is primarily mediated through the activation of SIRT1 enzymatic activity, which subsequently regulates various downstream targets via epigenetic mechanisms. High-mobility group box 1 (HMGB1) is a nuclear DNA-binding protein that, when acetylated, translocates to the cytoplasm and is released extracellularly. Once released, HMGB1 activates inflammatory responses by binding to toll-like receptor 4 (TLR4) and the receptor for advanced glycation end-products (RAGE) [17,18]. OA increases SIRT1 activity, which in turn deacetylates HMGB1, promoting its nuclear retention and thereby inhibiting inflammatory signaling [5]. SIRT1 deacetylates the p65 subunit of NF-κB, reducing its transcriptional activity and subsequently downregulating the expression of proinflammatory cytokines [19]. Based on the current literature, OA appears to mediate its antiinflammatory properties by activating SIRT1, thereby inhibiting NF-κB pathway activity [20].

Ongoing research regarding SIRT1 aims to elucidate the diverse roles of this protein within fundamental biological processes, to enhance our understanding of the ageing mechanism, and to explore its potential health-promoting effects. In parallel, numerous studies are investigating compounds that target SIRT1 activation for prospective therapeutic applications. In the present study, we aimed to evaluate the impact of OA upon SIRT1 expression by integrating computational analyses with experimental approaches.

## 2. Methods

### 2.1. Three-dimensional structure of sirtuin 1

The experimentally defined 3D structure of the NAD-dependent protein deacetylase SIRT1 used in the present study is available at various resolutions in the RCSB Protein Data Bank database. Most of these structures exist only excluding the C-terminal and N-terminal regions. The complexes with PDB codes BTR, 4I5I, and 4KXQ were not used, as they contain missing regions at the N-terminus and C-terminus and even within internal segments, which include regulatory domains and could potentially influence the outcome of the study. However, these structures were used for alignment to ensure that the substrate or compounds bind to the correct region. The 3D structure of the whole structure was chosen in order to increase the accuracy of the simulation studies with molecular dynamics (MD) methods, which were carried out in the later stages of the present study. It has been studied with the Q96EB6 access code structure (closed state) from the database Alpha Fold [21,22], which includes structures with high confidence and using artificial intelligence (AI), the favorite method in recent years. The regions of the structure that were identified as unreliable based on the “expected position error” were largely truncated, and the remaining parts were equilibrated using MD simulations. The 3D structures of resveratrol (CID 445154) and OA (CID 10494) to be docked in the allosteric site were obtained from PubChem. NAD^+^, 7-amino-4-methylcoumarin (AMC)-containing peptide and Zn^2+^ were extracted in the 5BTR, 4I5I, and 4KXQ complexes, which are the template structures obtained from the RCSB Protein Data Bank.

### 2.2. Assembling of ligands, cofactors, and protein

SIRT1 protein originally consists of 747 residues in total, and these regions are composed of NAD^+^, a Zn binding site, helical module, and C-terminal regulatory (CTR) segment. Before the ligands were docked to the protein, residues 1–161 from the C-terminal and 680–747 from the N-terminal were truncated, which would cause inconsistencies in the MD simulation. The binding sites of resveratrol, OA, AMC-containing peptide, NAD^+^, and Zn are based on 3D structures with access codes 5BTR, 4I5I, and 4KXQ from the RCSB Protein Data Bank. As a result of the alignment, resveratrol and OA were docked separately to the allosteric site, AMC-containing peptide to the substrate binding site, and finally NAD^+^ and Zn^2+^ to the binding sites.

### 2.3. Molecular dynamics simulation of complexes

Three different complexes were formed, the first of which was the complex that did not have any ligand bound to the allosteric site. The first of the other two complexes was the resveratrol-bound complex, which is known to have an allosteric effect in the literature [23], and the last was OA, the subject of the present study. Apo form (SIRT1-apo) and resveratrol (SIRT1-res)-bound forms of SIRT1 were designed as control groups, and the OA (SIRT1-ole)-bound form was designed as the subject of our study.

The ligands in the complexes used were resveratrol, OA, and NAD^+^; hence, it was necessary to define the force fields of these compounds before they could be simulated by MD methods. For this purpose, the parameters of atoms were defined with GAFF2 [24] force fields with the AMBER18 tool Antechamber [25]. In addition, semiempirical partial charges were assigned to atoms with the AM1-BCC charge method [26], and then the missing force field parameters were generated using parmchk2. With tLeap, another tool of AMBER18, the topology and coordinates of the complexes were produced, and the ff18SB [27] variant from AMBER force fields was used as the atom type. After defining the parameters such as atom and bond types, the complex was solvated with TIP3PBOX [28]-type water and neutralized with NACl in a 10-Å octahedral box.

These three complexes were energetically minimized to eliminate unfavorable interactions between atoms and to minimize high energy using AMBER18 [29], which is a suite of biomolecular simulation programs. The minimization was successfully completed using the steepest descent algorithm [30] in the first 10,000 steps and the conjugate gradient algorithm [31] in 90,000 steps. In the second stage, the system was heated to 300 K with Langevin dynamics (collision frequency γ = 10.0 ps^−1^) at constant volume and pressure for 1 ns with 1 fs steps, and the atoms were given an initial velocity. In this step, isotropic position scaling was used by restricting only hydrogen bonds in SHAKE mode. In the last step, all systems were simulated for 500 ns using an NPT ensemble with 2 fs steps. The simulation was run in a periodic box and the particle mesh Ewald (PME) method was used to calculate the electrostatic interactions in these systems. For this purpose, the dimensions of the charge grid were determined as 108 Å with the nfft1, nfft2, and nfft3 commands. In the present study, energy minimization was performed using a CPU-based computation with a Xeon 6258R 2.70 GHz processor, while molecular dynamics production simulations were carried out using an Nvidia V100 GPU.

### 2.4. MMGBSA free binding energy calculation

Molecular mechanics with generalized Born and surface area solvation (MM/GBSA) has been widely used to calculate the binding free energy of compounds to proteins. The end-state free energy method MMGBSA uses a generalized Born (GB) implicit solvent model. After the MD simulation was carried out in explicit solvent, the trajectory file was used, and snapshots were taken from this file at periodic intervals and studied in implicit solvent. In the present study, the estimated free binding energies of resveratrol and OA were calculated by taking snapshots every 200 frames at a salt concentration of 0.1 M and using the standard GB model [32]. In this calculation, the MMPBSA.py program [33], which is an AMBER18 tool and written in Python, was used.

### 2.5. Animal experiments

The 14 male Wistar rats utilized were obtained from the Experimental Animals Research Center at Atatürk University. All research procedures were sanctioned by the Local Ethics Committee for Animal Experiments at Atatürk University (2024/07-181). The rats were kept in clear polyethylene cages and given free access to a pelleted chow diet. The room was equipped with controlled conditions, including a temperature of 23 ± 2 °C, a 12-h light/dark cycle, and 55 ± 5% humidity, to allow 1 week of acclimation before the experiments began. In each experimental setup, 14 adult rats were randomly divided into two equal groups (n = 7 per group). The control group received 1 mL of distilled water daily via oral gavage throughout the experimental period and served as the placebo group. The treatment group was administered OA at a dose of 5 mg/kg body weight (Sigma Chemical Co., USA), also via oral gavage, once daily for 21 consecutive days. The selected dosage and route of administration were based on previous studies that have demonstrated the antioxidant, antiinflammatory, and tissue-protective effects of OA in various rodent models [34,35]. At the conclusion of the experiment, intracardiac blood samples and tissues were collected from the rats. Before sampling, the rats were anesthetized with ketamine (80 mg/kg; Ketalar, 50 mg/mL, Eczacıbaşı, İstanbul, Türkiye) and xylazine (10 mg/kg; Rompun, 2%, Bayer, İstanbul, Türkiye), and subsequently euthanized. The collected blood and tissue samples were then prepared for SIRT1 analysis.

### 2.6. Serum and tissue sirtuin-1 activity

SIRT1 activity was quantified using the enzyme-linked immunosorbent assay (ELISA). The ELISA procedure was carried out according to the standard protocol recommended by the kit manufacturer (Bioassay Technology Laboratory, Nanhu Dist, Jiaxing 314000, Zhejiang Province, P.R. China). This technique relies on the specific interaction between an antigen and its corresponding antibody. Briefly, the wells of the microtiter plate were initially coated with a capture antibody specific to the target protein. To prevent nonspecific binding, the remaining surface was blocked using a protein-based blocking buffer. Subsequently, the samples were added and incubated to allow antigen–antibody binding. After incubation, unbound substances were removed by washing. An enzyme-conjugated secondary antibody was then added and incubated to form a detection complex. Following another washing step, a substrate solution was introduced. The enzyme catalyzed a color reaction, and the intensity of the resulting color—measured spectrophotometrically at 450 nm—was directly proportional to the concentration of the analyte in the sample [36]. The OD value correlates with the concentration of SIRT1 in the rat samples, and the results are expressed in ng/mL.

### 2.7. Statistical analysis

The findings were analyzed using SPSS, version 21.0 for Windows. Prior to the analysis, the data were assessed for normality and homogeneity of variances to ensure the appropriateness of the parametric tests. Following verification of these assumptions, Student’s t-test was applied for comparisons between two independent groups in order to determine statistically significant differences in the parameters measured. A p-value ≤0.05 was regarded as statistically significant. The results are reported as mean ± standard deviation (SD).

## 3. Results and discussion

### 3.1. The structure and mechanism of SIRT1

**Figure 1:**
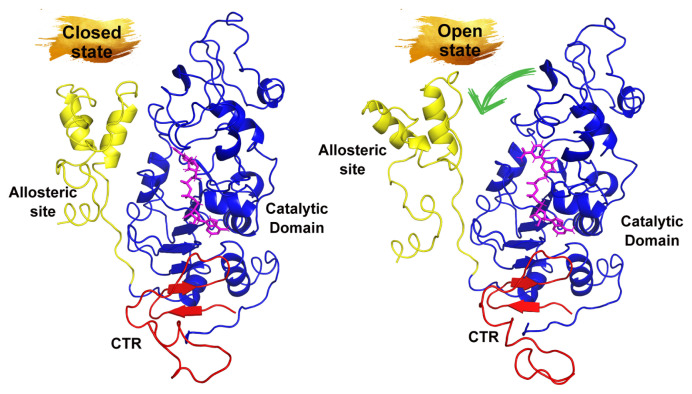
Three-dimensional representation of the open and closed conformation of SIRT1. Allosteric site, catalytic domain, and CTR are indicated in yellow, blue, and red, respectively. NAD+ is also shown in the catalytic domain of SIRT1 in magenta.

SIRT1 (human) consists of 747 residues and basically 4 domains. These are the zinc binding site, the catalytic domain, that is, the NAD binding site (244–512), the allosteric site (183–243), and the C-terminal regulatory (CTR) domain [37] (Figure 1).

**Figure 2:**
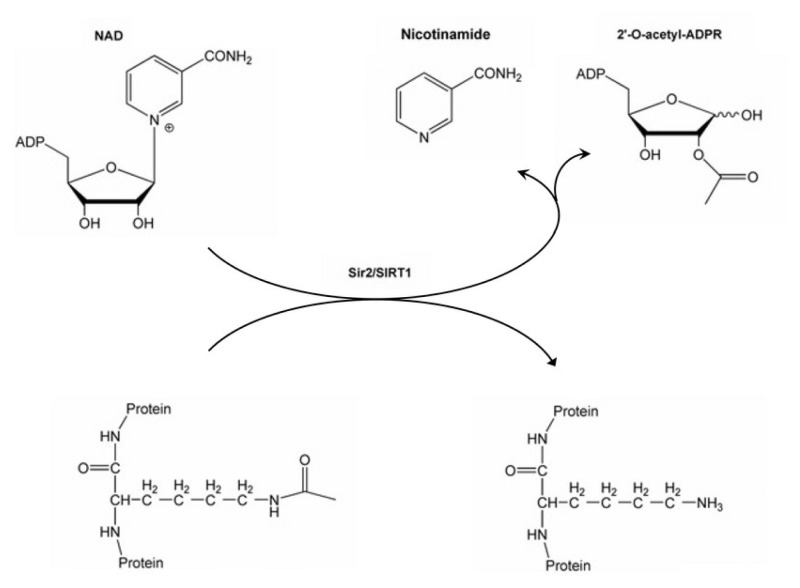
The NAD+-dependent SIRT1 deacetylase reaction.

SIRT1 performs its enzymatic activity in the presence of NAD, but Zn does not directly participate in the reaction. It only provides integrity and stability for the reaction to occur in the catalytic domain. The reaction takes place in the form of transferring the acetyl group in the substrate to the O-acetyl-ADP ribose group, which is formed as a result of the cleavage of NAD, and also provides the formation of a nicotinamide (NAM) group (Figure 2) [38,39]. NAM is also expressed as the negative regulator of SIRT1 [40]. The site focused on in the present study was the allosteric region of SIRT1, and this region is known in the literature as the N-terminal domain (NTD). Resveratrol, a compound with an allosteric effect, has been reported to bind to this region, increasing the affinity of substrates containing an acetyl group to the catalytic domain of SIRT1 [41,42]. It is also known from the resveratrol-bound SIRT1 crystal structures that the allosteric site approaches the catalytic domain [41].

**Figure 3:**
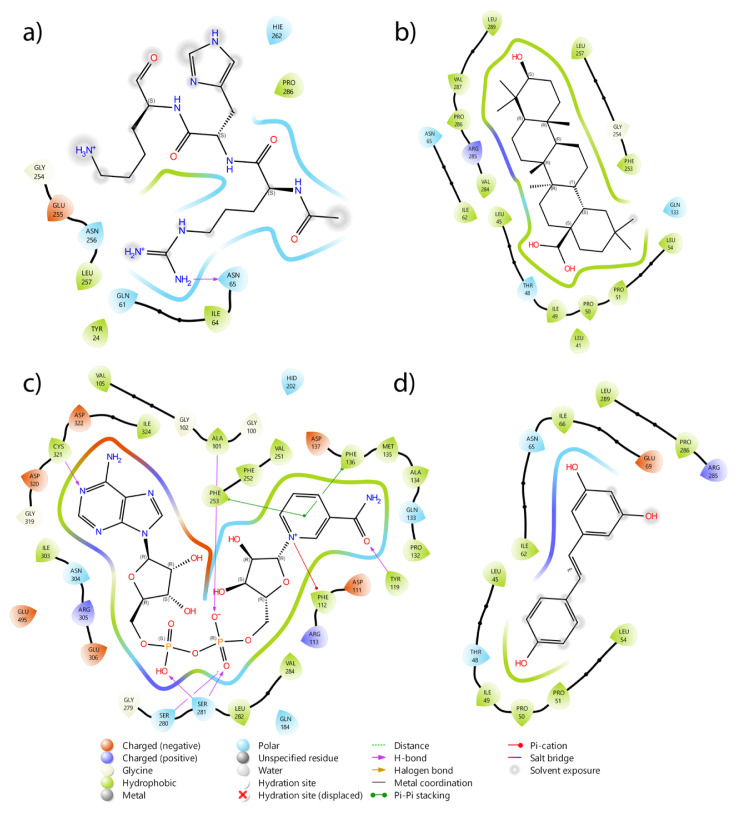
The binding modes of compounds: a) AMC-containing peptide, b) Oleanolic acid, c) NAD, d) Resveratrol (2D ligand–protein interactions were generated with Schrödinger Maestro).

Figure 3 presents the 2D binding interaction diagrams of the following compounds: AMC-containing peptide, OA, NAD^+^, and resveratrol. The diagrams illustrate the specific interactions between each compound and the SIRT1 active site, highlighting hydrogen bonds, hydrophobic contacts, and other relevant molecular interactions. These visualizations provide insight into the binding modes and the key residues involved in the ligand–receptor interface. Based on these binding modes, MD simulations were performed to evaluate the stability and dynamic behavior of the ligand–SIRT1 complexes.

### 3.2. Structural dynamics of the complexes

**Figure 4:**
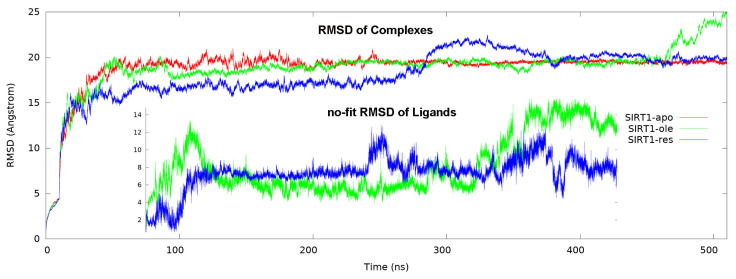
RMSD of complexes and ligands. Apo form, oleanolic acid-bound and resveratrol-bound are represented in red, green, and blue, respectively. In the inner graph, only the RMSD graph of the ligand is drawn and given comparatively.

The root-mean-square deviation (RMSD) calculation is a basic type of analysis that shows the dynamics of the protein throughout the simulation in computational studies. In this calculation, it gives very clear information about how the atomic deviation changes during the simulation. In the present study, three different complexes were formed: the apo form, the positive control group resveratrol-bound form, and the OA-bound form, which was the subject of the study. In Figure 4, the calculated RMSD values of these three complexes during the simulation were plotted. According to this graph, the apo form reached complete equilibrium after 100 ns and remained stable. Although the resveratrol-bound form had a lower RMSD value, it was determined that it reached equilibrium after showing an average of 5 Å jump after 300 ns. The reason for this mobility was understood to be not from the change in the total conformation of SIRT1; rather the movement of the AMC-containing substrate was found responsible for deviation. The plot shown in the inner graph is the RMSD of ligands only. As can be understood from this, resveratrol reached equilibrium after 100 ns and had a deviation of only 1–2 Å after that. When the RMSD graph of the OA-bound form was examined, it was determined that it reached equilibrium after 100 ns, but jumped after 450 ns. When looking at the reason for this mobility from the film formed during the simulation, it was found that it was also caused by the movement of the AMC-containing substrate. In the inner graph, it was noted that OA reached an increasing RMSD score of about 8 Å after 350 ns. When looking at the created film, it was found that OA was displaced towards the catalytic region.

**Figure 5:**
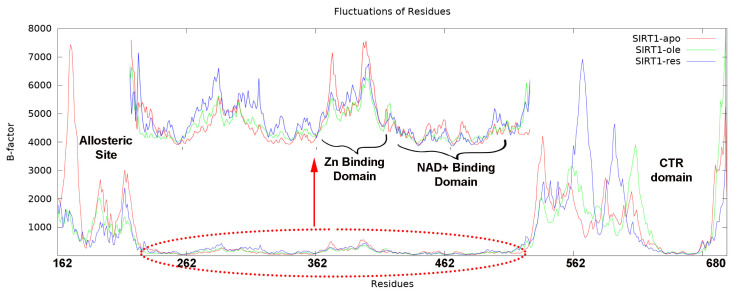
Residue fluctuation of SIRT1. Apo form, oleanolic acid-bound and resveratrol-bound are represented in red, green, and blue, respectively.

Another basic type of analysis is the root-mean-square fluctuation (RMSF) calculation. In this calculation, the deviations during the simulation can be examined locally by considering the residues and this calculation was taken into account in terms of the B-factor, which is the heat factor (Figure 5). When the allosteric region in the N-terminal domain was compared within itself, it was found that the apo form had the highest fluctuation and the resveratrol-bound complex the least. The same was true for the Zn binding site. No significant difference was calculated in the NAD^+^ binding site or the CTR domain. As can be seen from the graph, the region with the highest fluctuation was the site with the primary structure in the C-terminal region. It turned out that this region compacted towards the catalytic domain as the most displaced part during the simulation.

**Figure 6:**
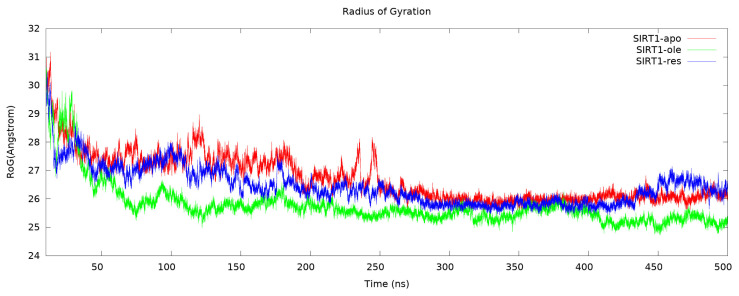
Cα radius of gyration (RoG) of all complexes during 500-ns MD simulation.

Another type of analysis that gave information about the dynamics of the protein was the radius of gyration. This calculation is a measure of the compactness of the protein structure, that is, the change around the axis of the atoms in the protein. In other words, it gives information about the structural activity of the protein [43]. In the present study, the radius of the gyration graph was drawn to compare the change in the dynamics of the protein after binding to SIRT1 with resveratrol and OA (Figure 6). According to this graph, all the complexes reached equilibrium at the end of 500 ns, but the complex with the highest gyration around itself of the atoms was revealed in the OA-bound complex. It was found that the apo form reached complete equilibrium, especially after 250 ns, and gave parallel results in the resveratrol-bound complex. These results were consistent with the RMSD and RMSF calculations. The conclusion to be drawn from this is that the effect of OA on the dynamics and compactness of SIRT1 is greater than that of resveratrol, but, eventually, it reaches equilibrium in both.

**Figure 7:**
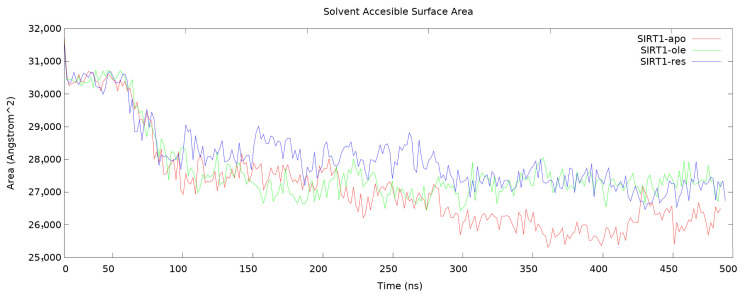
Solvent accessible surface area (SASA) of all complexes during 500-ns MD simulation.

A measure of protein folding and stability is the solvent-accessible surface area (SASA) calculation. The residues on the surface determine the areas that the solvent can reach on the surface of the protein [44–46]. Therefore, it is an important analysis in elucidating the structure–function relationship of protein. During the 500 ns simulation of the three different complexes in the present study, the protein’s SASA scores (Figure 7) were plotted. According to this graph, it was concluded that all complexes became compact and stable after 250 ns, but there were some differences. The SASA of the apo form reached lower levels than the other complexes. In addition, resveratrol and OA had similar SASA values and had a similar effect on the compactness of the protein. As a result, resveratrol and OA change the function of the protein and follow a similar pattern.

### 3.3. The mechanism of the allosteric site

**Figure 8:**
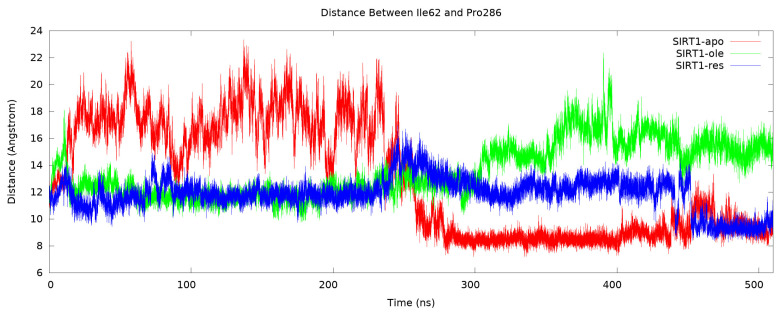
The distance between allosteric site and catalytic domain. It was calculated based on the distance between the αC’s of the Ile62 and Pro286 residues.

The focus of the present study was to examine the effect of OA on the structure of SIRT1 by comparing it with the reference molecule resveratrol when bound to the allosteric site. For this purpose, how the allosteric site moved and its effect on the catalytic domain were important criteria. To observe this movement for 500 ns, the distance variation between Ile62 on the allosteric site and Pro286 on the catalytic domain was first calculated (Figure 8). It was calculated based on the distance between the αC’s of the Ile62 and Pro286 residues. At the beginning of the simulation, SIRT1 was in a closed form, that is, the allosteric site was in the conformation close to the catalytic domain. When the graph was followed, it was found that the allosteric site in the apo form moved away from the catalytic domain in the first stage and started to approach the catalytic domain as of 250 ns and remained stable by docking completely by 300 ns. When the change in SIRT1 due to resveratrol, which is known to have an allosteric effect, was analyzed, there were differences. It turned out that the position of the allosteric site at the beginning of the simulation remained stable up to about 450 ns, and then it got closer to the catalytic domain. This distance was calculated as almost the same as that of the apo form. This stability of the allosteric site after resveratrol binding is suggested to increase the activity of SIRT1 [41,47]. Whether such a tight relationship between the allosteric domain and the catalytic domain was also present when OA was bound was compared by looking at the graph. As can be seen here, the allosteric site remained stable on the catalytic domain, just like in resveratrol, but with one difference. This difference was the outlier of the position of the allosteric site at the end of the simulation.

**Figure 9:**
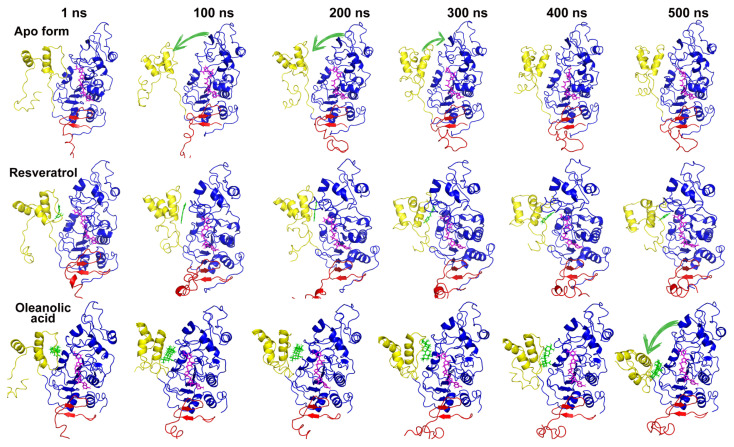
The mobility of allosteric sites in all complexes. Allosteric site, catalytic domain, and CTR are shown as yellow, blue, and red, respectively.

In Figure 9, how the movement of the allosteric site in all complexes changed was visualized comparatively in the timeline. In the figure, the allosteric site is shown in yellow and the catalytic domain in blue. When the apo form was examined, it was seen that the allosteric site first moved away from the catalytic domain, then approached it, and became compact. On the other hand, in resveratrol-bound SIRT1 it was observed that it was completely compact and even had a tighter relationship at the end of the simulation. In the distance analysis, it was stated that these two regions, like resveratrol in OA, were in close interaction, but there was a small discrepancy in the graphic pattern. As can be seen from the 3D visualization, the allosteric domain remained compact on the catalytic domain in OA-bound SIRT1. However, at the end of the simulation, while the allosteric site remained compact, there was a noticeable change in its orientation, which could potentially affect the protein’s function. Even small conformational shifts in protein structures can lead to significant biological consequences. Therefore, it was concluded that this tight relationship, which increases the activity of SIRT1, continues even when docked to OA.

### 3.4. The estimated binding energy of the ligands

**Figure 10:**
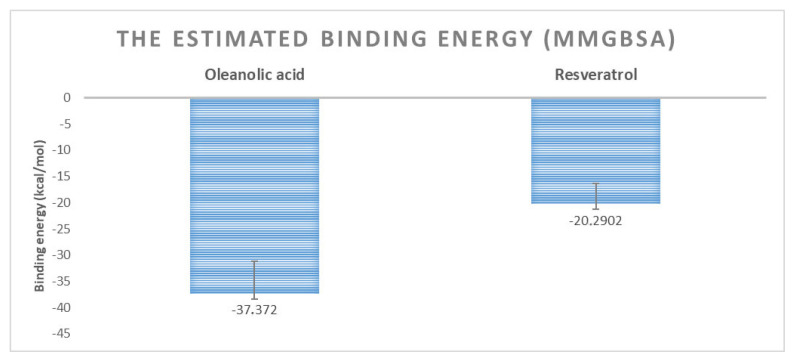
The estimated binding energy of oleanolic acid and resveratrol to SIRT1.

The MM-GBSA estimated binding energy calculation is a measure of the binding affinity of ligands to macromolecules [48]. The MM-GBSA binding energy calculation is an effective method for comparing the binding affinities of ligands within the same dataset. Moreover, this postsimulation binding energy calculation provides more accurate results compared to those derived from molecular docking studies [49,50]. In the present study, the comparative binding affinities of resveratrol and OA for the SIRT1 allosteric site were evaluated using this approach. The estimated free binding energy of resveratrol is –20.29 kcal/mol (SD = 3.89; SEM = 0.12), while that of OA is −37.37 kcal/mol (SD = 6.21; SEM = 0.19) (Figure 10). According to these results, it was concluded that the binding affinity of OA to the allosteric site of SIRT1 is higher than that of resveratrol and that it will be a promising compound.

### 3.5. SIRT1 activity in rat serum and liver and kidney tissue

In our research, we conducted experimental studies to explore the impact of OA on SIRT1 in liver and kidney tissues, as well as in serum samples. Our results clearly demonstrated (Table) that serum SIRT1 activity was higher in the OA-treated rats (23.88 ± 0.80) compared to the control group (10.55 ± 0.93) (p < 0.001). Nutraceutical combinations that enhance SIRT1 activity through various contributing processes may hold significant possibilities for promoting health. Different formulations of food-based products thought to enhance SIRT1 activity by possibly supportive or enhancing pathways may be assessed in preclinical studies to identify those most suitable for clinical investigation. The activity of SIRT1 could offer various benefits to liver health, making it valuable to explore natural compounds that activate SIRT1 within the framework of hepatotoxicity research [51]. One of the most significant bioactivities of OA is its protective effect against liver toxicity [52]. In the literature, OA has been utilized in rats with experimentally induced liver damage, demonstrating a substantial protective effect against hepatotoxicity. It has been observed that OA brought serum enzyme levels, indicative of hepatotoxicity, back to normal [53]. SIRT1 plays a crucial role in regulating the body’s response to inflammation in the liver and it has been reported that OA treatment increases SIRT1 levels [54]. In our study, SIRT1 activity in liver tissue was greater in the OA-treated rats (20.69 ± 0.51) compared to the control group (11.41 ± 1.23) (p < 0.001). Our study results suggest that OA provides hepatic protection by promoting the upregulation of SIRT1.

In the literature, it has been documented that SIRT1 significantly contributes to maintaining and enhancing kidney health through various mechanisms. SIRT1 may reduce inflammation in kidney tissue, thereby helping to protect the kidneys from inflammatory conditions. Another protective mechanism of SIRT1 in the kidney is autophagy. By regulating the process of autophagy, it can stimulate cells to undergo self-cleaning. By facilitating the removal of damaged cells and toxins, it may help preserve kidney health [55,56]. In the kidney, SIRT1 is widely expressed in tubular cells and podocytes. Our results suggest that the kidney SIRT1 activity was higher in the group treated with OA (16.14 ± 1.64) compared to the control group (10.29 ± 1.23) (p < 0.05). When evaluating kidney outcomes, it appears that OA, as a potent SIRT1 activator, could represent an original health-promoting target for the protection and remediation of renal diseases.

## 4. Conclusion

In the present study, we investigated the potential of OA as a candidate compound with allosteric effects on SIRT1, comparing it to the well-established SIRT1 activator resveratrol. The 3D structure of SIRT1 was modeled using AI methods, with a high confidence rate (>90%) based on current AI techniques widely utilized across various scientific fields. The binding interactions of NAD+, Zn, AMC peptide, resveratrol, and OA were assessed by docking these ligands into their respective binding sites, using crystal structure data obtained from experimental databases. To enhance the precision of the findings, we performed a 500-ns MD simulation, which provided insights into the dynamic behavior of the protein–ligand complexes over time. Additionally, MM-GBSA energy calculations were conducted to evaluate the binding affinities of these compounds, offering a deeper understanding of their potential allosteric interactions with SIRT1. This approach allows for a more reliable comparison of the binding energies, particularly when compared to docking studies alone.

The results of our simulation and energy calculations revealed that both resveratrol and OA were able to stabilize and equilibrate at the SIRT1 allosteric site by the end of the simulation. Upon binding, both compounds induced significant changes in the 3D structure of the allosteric site, with a noticeable reorientation towards the catalytic domain, resulting in a more compact and stable structure. This structural change was particularly evident for OA, which exhibited a higher binding affinity to the allosteric site compared to resveratrol. The enhanced binding affinity of OA suggests that it may exert an allosteric effect similar to that of resveratrol, thus potentially increasing SIRT1 activity [57]. These computational findings were corroborated by experimental data, which demonstrated that OA significantly increased SIRT1 expression in serum and liver and kidney tissues. Collectively, these results highlight the therapeutic potential of OA as a natural activator of SIRT1, with implications for modulating inflammation, oxidative stress, and antioxidant capacity in liver and kidney diseases. Future studies should focus on optimizing the pharmacokinetic properties of OA and conducting well-designed clinical trials to evaluate its safety, efficacy, and possible synergistic effects with other SIRT1 activators or agents targeting related pathways [58].

## Figures and Tables

**Table:** Effect of oleanolic acid treatment on serum and liver and kidney tissue SIRT1 activity.

	Parameters		
Groups	Serum SIRT1 (ng/mL)	Kidney SIRT1 (ng/mL)	Liver SIRT1 (ng/mL)
**Control**	10.55 ± 0.93	10.29 ± 1.23	11.41 ± 1.23
**Oleanolic acid**	23.88 ± 0.80^a^	16.14 ± 1.64^b^	20.69 ± 0.5^1^a

Data are the least square means ± SD.

a Significantly different when compared with a control group (p < 0.001).

b Significantly different when compared with a control group (p < 0.05).

The treatments were applied for 21 days. Control group = rats given 1 mL of distilled water via oral gavage, Oleanolic acid group = 5 mg/kg oleanolic acid (dissolved in distilled water) by oral gavage.

## Data Availability

The datasets analyzed during the current study are available in the PubChem repository. The specific data can be accessed via the following links: https://pubchem.ncbi.nlm.nih.gov/compound/445154 https://pubchem.ncbi.nlm.nih.gov/compound/10494
